# Production and partial purification of thermostable bacteriocins from *Bacillus pumilus* ZED17 and DFAR8 strains with antifungal activity

**DOI:** 10.22099/mbrc.2019.31563.1367

**Published:** 2019-03

**Authors:** Shahrzad Dehghanifar, Mehrnaz Keyhanfar, Giti Emtiazi

**Affiliations:** 1 Department of Biotechnology, Faculty of Advanced Sciences & Technologies, University of Isfahan, Isfahan 81746-73441, Iran; 2 Department of Biology, Faculty of Sciences, University of Isfahan, Isfahan, 8146-73441, Iran

**Keywords:** *Bacillus*, Bacteriocin, Antifungal, *Rhizoctonia solani*, Biocontrol

## Abstract

The bacteria which are members of the genus *Bacillus* are known to produce a wide variety of antimicrobial substances and bacteriocins. The main objective of this study was to investigate the effect of these bacteriocins on eukaryotic cells such as fungi, yeast and plant seeds. Several strains were screened for antifungal activities and identified by the means of polymerase chain reaction (PCR) of the 16s rRNA gene and sequencing. Our experiments showed that the *Bacillus pumilus* ZED17 and DFAR8 strains, had antifungal activities against *Rhizoctonia solani* and selected for further investigations. Extracellular peptides produced by these strains were purified by ammonium sulfate precipitation and dialysis. Addition of these peptides to Potato Dextrose Agar (PDA) medium inoculated with *R. solani* indicated significant inhibition of the fungal growth. The antifungal peptides were thermo-stable and remained active after boiling at 100˚C for 15 min. The molecular weight of the peptide with antifungal activity was estimated by electrophoresis on the Sodium Dodecyl Sulfate Poly Acrylamide Gel (SDS-PAGE) as about 5 KDa. Structural nature of this peptide was determined after gel extraction by Fourier-transform infrared spectroscopy (FTIR). Moreover, this peptide showed inhibiting effect on seeds germination of some herbs. This peptide could be applied to control herbal fungal disease induced by *R. solani* which is a broad host range plant pathogen fungus and its inhibition is very important. The peptide also prevents seed germination. Hence, it can be appropriate for inhibiting weeds growth. No significant effect against *Saccharomyces cerevisiae* and *Candida albicans* was observed.

## INTRODUCTION

Bacteriocins are natural peptides secreted by many varieties of bacteria for the purpose of destroying other organisms [1] and are mostly active against closely related species [2]. This capability of the bacterial strains contributes to the elimination of competition, for access to resources in their environment [1]. All bacteriocins are ribosomally synthesized peptides that are categorized based on producer organism, molecular size, chemical structure and mode of action [3]. Bacteriocins exert their effects by a variety of mechanisms like pore formation in the membrane [1]. Effective communication of the bacteriocins with the plasma membrane of sensitive cells is indicative of their lethality [4]. Because the main primary target of the bacteriocins is the plasma membrane of the cells [5], most bacteriocins have common characteristics like low molecular weight, heat stability and the cationic and hydrophobic nature [3]. Their positive charge simplifies the interaction with negative phospholipids membranes and leads to membrane permeability [6] and depletion of the proton motive force of the target cells [7]. 

Bacteriocins were first recognized in 1925 by Andre Gratia when he observed that the growth of various bacteria strains was inhibited by a substance with antibacterial properties which was called Colicin V [3]. For several reasons the bacteriocins are probable substitutes for antibiotics. For instance, they are active at low concentrations and could be metabolized by the recipient [1]. Bacteriocins have other interesting features: they are easily produced through natural resources, and having a wide range of activities. In addition, these compounds are not susceptible to recipient resistance [8]. The emersion of multidrug resistant pathogens and limited use of antibiotics as feed additives led to an intensive search for new suitable alternatives [9]. 

Members of genus *Bacillus* are found in diverse environments, which are Gram-positive, aerobic, rod-shaped and endospore-forming bacteria [10]. They can be applied in generating compounds like amylase, proteases, antibiotics, and surfactants [11]. The antagonistic effect of *Bacillus* is due to secondary metabolite production like antibiotics and antimicrobial peptides [12]. The compounds are produced in the early stationary growth phase and their quantity is influenced by composition of the culture medium, host microorganism, temperature, pH and incubation conditions [13]. Beacause of sporulation, variation in morphological characteristics and production of antimicrobial substances, the *Bacillus* strains are able to survive in various conditions [10]. Due to high level production and direct release of the peptides into the extracellular space, *Bacillus* strains are suitable organisms for the production of these antimicrobial compounds. In addition, protein secretion may create better folding conditions in comparison with an intracellular reducing environment by preventing the formation of inclusion bodies. Moreover the separation of protein from the other components will be easier [14]. 

Today, resistance to all clinically used antibiotics has reached a point where the World Health Organization (WHO) has expressed concern about the possibility of resistant pathogens becoming incurable [8]. Fungal strains can be pathogenic for human and cause a variety of diseases such as dermatophytosis, candidiasis, aspergillosis, histoplasmosis and cryptococcosis in normal and immunosuppressed hosts [15]. In addition, fungi are the most abundant group of plant pathogens. Some fungal species including *Fusarium*, *Nigrospora* and *Rhizoctonia* are involved in plant diseases and cause reduction in yields of important agricultural products such as rice, wheat and potato [16, 17].

Moreover, a serious economic issue for the pulp and paper industry is decaying of the stored wood contaminated with various fungal infections [13]. Many plant diseases are caused by bacteria, viruses and fungi that greatly reduce the quality and safety of agricultural products are controlled by chemical pesticides and fungicides [18]. These pesticides have high toxicity, allergenic properties [13] and unfavorable environmental effects like the emergence of resistance against fungicides and the dispersion of their residues in the food chain [18]. To overcome these deficiencies, many countries around the world use biological control rather than chemical control for plant diseases [19]. Common antifungal compounds produced by *Streptomyces*, such as amphotericin and nystatin have restrictions such as narrow range spectrum, low permeability to specific tissues, and toxicity for the human body [20]. Thus, more studies must be conducted to discover and evaluate new compounds as an alternative to current antibiotics and pesticides. The growth inhibition of fungal pathogens including *Sclerotinia*, *Fusarium*, *Gaeummanomyces*, *Nectria*, *Pythium* and *Phytophthora*, *Aspergillus flavus*, *Aspergillus niger* by some strains of *Bacillus* such as *B. cereu*s, *B. subtilis*, *B. mycoides* are reported [13, 21]. Some peptides with antifungal and antibacterial properties, produced by the genus *Bacillus *include mycobacillin, iturin, bacillomycin, surfactin, mycosubtilin, fungistatin, subsporin [21] cerecin7, Tochicin, thuricin 439, entomocidus9 [22], subtilin and subtilosin [23]. Many studies are conducted on the antibacterial effect of these compounds. 

The objective of this study is to examine the effects of the peptides derived from the *Bacillus* strains on eukaryotic cells like the yeasts, fungus and plant seeds. *Rhizoctonia* is a plant pathogen fungus with a broad host ranges including monocot and dicot plants with economic importance such as rice, wheat, alfalfa, beans, soy, corn, potato and tomato; therefore the inhibition of these fungus is very important.

## MATERIALS AND METHODS

**Bacterial strains and culture conditions: **Nine bacteria were isolated from soil and rhizosphere of different plant based on a method used by Shokri and Emtiazi, and identified as *Bacillus* through biochemical characteristics using standard methods [24]. The obtained results were confirmed based on 16S rRNA PCR by Takapozist Company (Tehran, Iran), where PCRs were performed using DNAs extracts with universal primers RW01 and DG74 that their sequences are listed in the next section. The *Bacillus* and Saccharomyces cerevisiae strains were cultured in nutrient agar medium at 37˚C. The *R. solani*, *Mucoraceae* and *Candida albicans* strains were grown in PDA (Potato Dextrose Agar) medium at 30˚C, for three days.

**Isolation of bacteria with antifungal activity: **Antimicrobial activity is determined by the spot on lawn method against different fungi and yeasts. The *Bacillus* strains were tested for their ability to inhibit the growth of various fungi. These strains were grown in 100 ml nutrient broth at 37˚C for 24 h subject to 80 rpm shake. The cells were removed through centrifugation (1610×g, 15 min) and the supernatant is refined through a 0.45-mm filter. The strains of fungi including *R. solani*, *Mucor rouxii  DSM1194*  and the strains of yeasts including *Candida albicans* and *Saccharomyces cerevisiae* were cultured by sterile swabs on PDA medium and a 50 µl of the filtered supernatant was added followed by incubation at 30˚C for 24 h simultaneously. 

In another experiment, 1×1 mm pieces of each fungi strain were inoculated into 100 ml of nutrient broth medium and 200 µl of the bacterial filtered supernatant (obtained as above) was added to nutrient broth medium inoculated with the fungus, followed by incubation for 7 days at 30˚C and subjected to 80 rpm shake. The fungal dry weight was then calculated. This experiment was performed in triplicate and one specimen including the inoculated medium with fungus without adding the bacterial filtered extract, considered as the control.

**Identification of bacteriocin producing isolated bacteria: **In order to identify the selected *Bacillus* strains with the best antifungal activity, the strains were grown on nutrient agar medium and incubated at 37˚C for 24 h. The DNA extraction was performed through boiling the bacterial suspension in sterile distilled water at 100˚C for 10 min and centrifuged (1250×g, 15 min). The supernatant was used as a DNA template [25]. The PCR is performed through the 16S rRNA universal primers RW01 (5′-AAC TGG AGG AAG GTG GGG AT-3′) and DG74 (5′-AGG AGG TGA TCC AAC GC A-3′) under the following conditions: 94˚C for 5 min, 30 cycles of (94˚C for 45 seconds, 54˚C for 30 seconds, 72˚C for 5 min, 72˚C for 10 min). The PCR amplified DNA was electrophoresed on 1.5% agarose gel with the DNA molecular weight marker (100-1500bp DNA ladder, CINNAGEN, IRAN) and visualized after staining with DNA Safe Stain (CINNAGEN, IRAN). Water as a negative control and the DNA template as a positive control of PCR were considered .The result of sequence analysis was compared to the data from the National Center for Biotechnology Information, MD, USA (NCBI) Gene Bank with nucleotide BLAST.

**Identification of Bacteriocin producing gene in** ***Bacillus*** **strains: **To determine the types of produced bacteriocins, the PCR is performed on 3 *Bacillus* strains using bacilysin primers produced by Takapozist Company (Tehran, Iran), under the following conditions: 95˚C for 10 min, 30 cycles of (94˚C for 1 min, 59˚C for 1 min, 72˚C for 2 min and 72˚C for 10 min). In addition, the PCR is performed on these strains under temperature gradient. The primers (BACAB-F) 5′-CTT CTC CAA GGG GTG AAC AG-3′ and (BACAB-R) 5′-TGT AGG TTT CAC CGG CTT TC-3′) were used [26].

**Production and extraction of antifungal compounds:** *Bacillus* strains are cultured in 200 ml nutrient agar and incubated at 37˚C under aeration for 24 h. The cells were removed by centrifugation (1610×g, 15 min) and the supernatant is filtered through 0.45 µm membranes, and then precipitated with ammonium sulfate to 60% saturation. Then, the saturated solution incubated at 4°C for 24 hours and centrifuged (14500 ×g for 30 min) .The resulting pellets were dissolved in phosphate buffer with pH 7.0 and dialyzed against the same buffer through dialysis tube of 1000 Da. The partially purified compound was tested for antifungal activity by adding 100 µl of this compound simultaneously to an indicator strain of fungi (*R.solani*) cultured on PDA medium and incubated at 30˚C for 24 h.

**Molecular weight determination and detection of antifungal activity of purified peptide: **To determine the molecular weight of this partially purified antifungal compound, the SDS-PAGE was carried out followed by Zymography techniques Which is an electrophoretic technique to estimate the relative location of the studied peptide (details in **Fig 6**). Electrophoresis is conducted at 100V for 2 h. The gel was then sectioned into two parts: one contained a pre-stained protein marker (Chromatein Pre-stained Protein Ladder-vivantis Technologies PR0602) which was stained with Coomassie Brilliant Blue, and the other was studied for direct antifungal activity. To maintain the natural conformation of the proteins, the gels were placed in Triton X-100 for 30 min, washed with sterile distilled water at every 30 minutes for 2 hours and then overlaid on PDA medium inoculated with indicator strain *R. solani* and incubated at 30˚C [27].

**Extraction of antifungal peptide from gel and analyzing the petide by FTIR: **Antifungal compounds of the two *Bacillus* strains were subjected to electrophoresis as mentioned above. Sections of the gels that contain the anti-fungal activity were cut horizontally with a sterile scalpel and then 3 ml of elution buffer containing NaCl 150 mM, EDTA 1 mM and Tris-HCL 50 Mm was added on them. The mixture was crushed with a sterile glass rod and incubated overnight at 30˚C under stirring. The mixtures were then centrifuged (1250×g, 10 min). Supernatants containing partially purified peptides with antifungal activity were studied again to make sure the activity was maintained. 100µl of the purified peptides was added to PDA medium which contained an indicator strain of fungi (*R. solani*). The purified peptides from polyacrylamide gel were freeze-dried and analyzed with a (JASCO 6300 JAPAN) Fourier transform infrared (FTIR) spectrophotometer, to determine the natural structure of these peptides.

**Investigating the effect of antifungal compound on seeds germination: **To study the effect of antifungal compounds on seeds germination, the *Raphanus sativus* and *Lepidium sativum* seeds were first sterilized as follows: seeds were placed in 70% ethanol for 1 min and washed with distilled water. Sodium hypochlorite was diluted with distilled water with 20 to 80 ratio and the seeds were placed in it for 3-5 minutes and washed 3 times for 2-3 minutes in sterile distilled water. These sterilized seeds were cultured in Murashige and Skoog (MS) medium containing 100 ml macro elements included nitrogen, phosphorus, potassium, calcium, sulfur, magnesium, carbon and 10 ml micro element such as boron, chlorine, manganese , zinc , copper, molybdenum , nickel , 10 ml iron, 10 ml vitamin, 10 gr sugar and 30 gr agar. The purified bacteriocins through ammonium sulfate precipitation and dialysis were added to the seeds in MS medium and then incubated at 30˚C exposed to light for 24 h. Experiments were performed in triplicate and a sample was considered as the control specimen, then the germination rates were measured and compared with the control. 

**Investigating the effect of temperature on activity of antifungal compound: **The partially purified bacteriocin was examined to determine the thermal stability. The obtained solution with antifungal activity was apportioned into six vials, and each vial becomes subject to temperatures of -20, 0, 4, 37, 70 and 100˚C for 15 min. The activity was evaluated and 100 µl of each of the portions was added to the culture medium inoculated with the fungi and then incubated for 24 h at 30˚C.

## RESULTS

In this study, bacterial strains showed inhibition effects against *R. solani* and *Mucor rouxii* (DSM1194) with a visible growth inhibition zone. However, their effects against *Saccharomyces cerevisiae* and *Candida albicans* were insignificant. Moreover, the degree of growth inhibition of yeasts and fungi were tested through direct antagonism on plates by adding the filtered supernatant to the liquid medium inoculated with the fungus followed by fungal dry weight calculation. 

The obtained results indicated significant inhibition of the fungal growth compared to the control sample. The results of triplicate experiments were analyzed by applying the Duncan test at the confidence level of 0.05% by SPPSS 6.0 software. This can determine whether differences were statistically significant. Among the 9 *Bacillus* strains, *B. Pumilus ZED17* (0.13±0.06) and *DFAR8* (0.12±0.06) showed the best antifungal effect and the best performance was against *R. solani* (0.91±0.15). Therefore, in this study the focus was on these strains. Identification of isolated bacteria producing bacteriosins by sequencing the PCR product, a new strain of the genus *Bacillus pumilus* was identified. The result of sequence analysis was compared to the data from the (NCBI) Gene Bank with nucleotide BLAST. 

**Figure 1:**
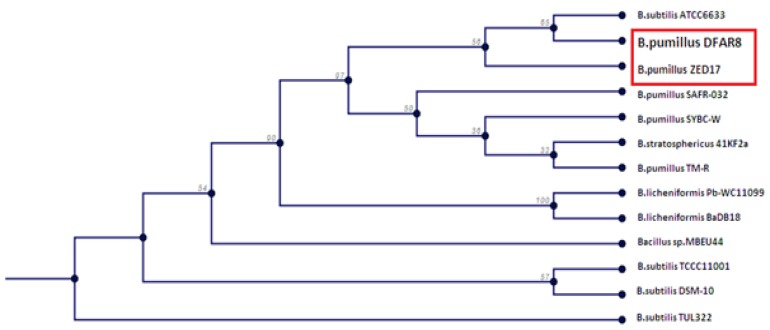
phylogenetical tree of *B.pumilus DFAR8* and *B.pumilus ZED17*. The tips of the tree represent groups of descendent species and the nodes on the tree represent the common ancestors of those descendants

The phylogenetic tree that was drawn by CLC sequence viewer 6.1.A phylogeny software represents the evolutionary relationships of this strain with 99% homology (Fig. 1). This strain is named *Bacillus pumilus DFAR8* with accession number of KC577596 which is recorded in the NCBI database.

Since bacilysin is found in many strains of *Bacillus*, PCR performed on B*. pumilus DFAR8* and *B. pumilus ZED17 *strains using bacAB gene primers that encode the bacilysin. The PCR analysis showed that this gene is not found in *Bacillus* strains. Therefore compound produced by these strains can be a bacteriocin other than bacilysin.

Antifungal activity of the partially purified compounds was tested after precipitation by ammonium sulfate and dialysis. 100 µl of the purified compounds was added to the indicator strain of fungi, *R.solani *cultured on PDA medium simultaneously. After incubation at 30˚C for 24 h, a growth inhibition zone became visible.

**Figure 2:**
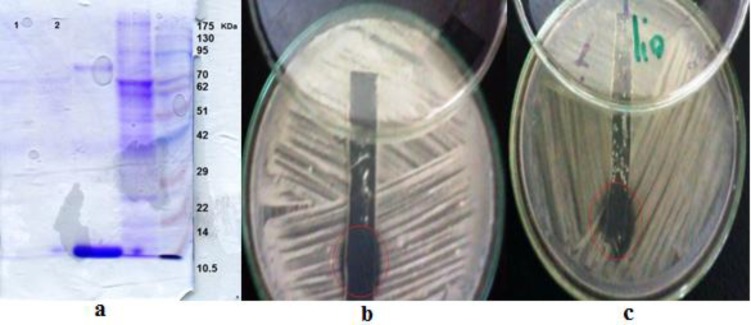
Gel electrophoresis analysis of antifungal peptides derived of Bacillus strains submitted to SDS-PAGE and stained for proteins with Coomassie blue (a) antifungal peptide derived from Bacillus strains from wells 1 and 2, and protein marker from well 5 were loaded on the gel. The peptide derived from B.pumilus ZED17 (b) B.pumilus DFAR8 (c) in unstained gel tested for antifungal activity on R. solani. In images b and c, the visible inhibition zones were observed in the cycles

The molecular weight of the partially purified antifungal compound was estimated through SDS-PAGE. The gel stained with Coomassie Brilliant Blue and the molecular weight of the compounds derived from *B. pamilus* *ZED17* and *B. pamilus DFAR8* were determined as<10 KDa (Fig. 2a). Antifungal activity could be revealed by overlaying the other part of the gel containing the same compound in media including the indicator strain *R.solani*, which showed a clear inhibition zone at the same region that was visible in the stained gel after 24 h at 30˚C (Fig. 2b,c).

The peptides extracted from the gel were added to the PDA medium inoculated with the fungi and a growth inhibition zone becomes visible after 24 h. 

**Figure 3:**
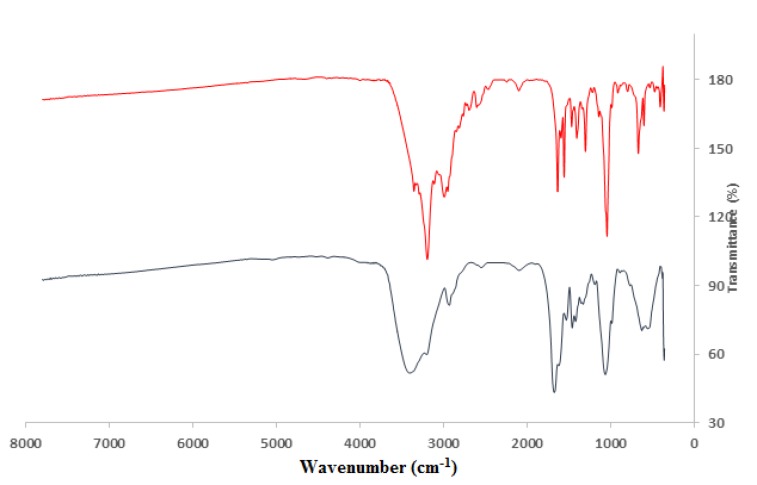
Fourier transform infrared (FTIR) spectrum of antifungal peptides derived from (upper line) Bacillus pumilus DFAR8 and (lower line) Bacillus pumilus ZED17 strains. The infrared spectrum of antifungal compound were drawn with Excell 2012 software and showed characteristic absorption valleys at 1671,1616,1630, 1553, 1296 cm-1 indicating the secondary structure of peptides include α-helix, β-sheet and turn structures. Valleys that result from C-H stretching (2928 cm-1) and (3197 cm-1) indicate the presence of aliphatic and aromatic chain respectively. The valley absorbance at 3999 cm-1 represents strong H-bonding of acid, amine & amid groups, and the valley at 3350 cm-1 is related to NH stretching of terminal amine

Samples extracted from the gel were freeze dried and analyzed with FTIR. The infrared spectrum of antifungal compound exhibited the characteristic absorption valleys at 1671,1616,1630, 1553, 1296 cm^-1^ indicating that the secondary structure of peptides include α-helix, β-sheet and turn structures (Fig. 3). Valleys formed through C-H stretching (2928 cm^-1^) and (3197 cm^-1^) indicate the presence of aliphatic and aromatic chains respectively. In addition, the absorption valley at 3999 cm^-1^ represents strong H-bonding of acid, amine and amid groups, while the valley at 3350 cm^-1^ is related to NH stretching of terminal amine.

**Figure 4:**
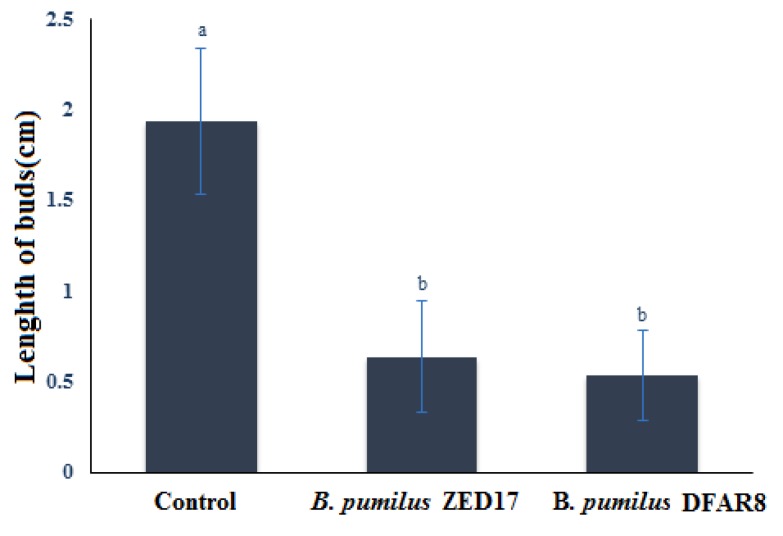
Inhibition of seeds germination after addition of partially purified bacteriocin obtained from Bacillus strains. Bars are means ±SD of triplicates. The P values were calculated by comparing the data with control using Duncan test at the confidence level of 0.05% by SPSS 6.0 software

Adding partially purified bacteriocin to sterilized seeds cultured on MS medium showed inhibition effect on seed germination compared to that of the control. Results of triplicate experiments are analyzed by applying the Duncan test at the confidence level of 0.05% by SPPSS 6.0 software. Different letters indicate significant difference with control sample (Fig. 4). The results indicated significant inhibition of seeds germination compared to the control sample. This peptide is suitable not only for pathogenic fungi but can be used for inhibiting weeds growth. 

The antifungal peptide remained active after exposure to different temperatures -20, 0, 4, 37, 70 and 100˚C. It was also significantly thermo stable, even after boiling at 100˚C for 15 min, as no changes were observed in the antifungal activity.

## DISCUSSION

The *Bacillus* strains produce secondary metabolites such as antibiotics, antifungals and siderophores. This ability makes them to be considered as an appropriate biological control agents [28]. In this study, antifungal compounds produced by *B.pumilus ZED17* and *B.pumilus DFAR8* are purified and characterized and their effect on eukaryotic cells was evaluated. Antifungal peptides are extracted by precipitation and dialysis process respectively. The molecular weight of compounds is determined to be <10 KDa by SDS-PAGE. Unstained gel in the same area showed antifungal activity. Peptides derived from this gel regions are relatively pure and are analyzed with FTIR. The FTIR provides additional information about the nature of the peptides. Analysis of the FTIR spectrum showed absorption bands related to the presence of the secondary structure of the peptide, CH stretching of aromatic ring, CH group aliphatic, strong H-bonding of acid, amine and amid groups and NH stretching of terminal amine in antifungal peptide. Perhaps since the large number of double bonds are found in the peptide of B.pumilus ZED17 strain, this peptide displays more complex folding condition. 

 The PCR analysis of bacAB genes related to bacilysin revealed this gene is not observed in *B.pumilus ZED17* and *B.pumilus DFAR8* strains. Therefore, to identify produced bacteriocin in these strains, the PCR can be conducted for the other bacteriocins genes with low molecular weight such as subtilin with molecular weight of 3.3 KDa or mersacidin with molecular weight of 1.8 KDa [3].

Moreover, the antifungal peptides are thermostable and remain active after boiling at 100˚C for 15 min. This compound prevents seed germination, meaning that it can also be effective in inhibiting weed growth. The obtained result confirmed the effect of peptides derived from *Bacillus* strains on the eukaryotic cells such as fungi and seeds of plants. The consumed antifungal compounds do not serve the purpose at all times, and might create recipient resistance while being toxic for human. As the members of Genus *Bacillus* can produce a variety of compounds with biological activity running more investigations on them can be considered as further tasks for researchers.
